# Immune Checkpoint Inhibitors in Epithelial Ovarian Cancer: An Overview on Efficacy and Future Perspectives

**DOI:** 10.3390/diagnostics10030146

**Published:** 2020-03-07

**Authors:** Fulvio Borella, Eleonora Ghisoni, Gaia Giannone, Stefano Cosma, Chiara Benedetto, Giorgio Valabrega, Dionyssios Katsaros

**Affiliations:** 1 Dionyssios Katsaros, Gynecology and Obstetrics 1, Department of Surgical Sciences, University of Turin, 10126 Turin, Italy; fulvio.borella87@gmail.com (F.B.); stefano.cosma@unito.it (S.C.); chiara.benedetto@unito.it (C.B.); 2 Department of Oncology, University of Torino, 10060 Torino, Italy; eleonora.ghisoni@ircc.it (E.G.); gaia.giannone@ircc.it (G.G.); giorgio.valabrega@ircc.it (G.V.); 3 Candiolo Cancer Institute, FPO—IRCCS, 10060 Candiolo, Italy

**Keywords:** Ovarian cancer, immunotherapy, immune checkpoint inhibitors, safety, biomarkers, clinical trials

## Abstract

Epithelial ovarian cancer (EOC) is the leading cause of death among gynecological cancers. Despite improvements in medical treatments, the prognosis for EOC remains poor, and there is an urgent need for new therapeutic strategies. Immune checkpoint inhibitors (CPIs) have dramatically improved survival of several cancers and are under evaluation in OC. Unfortunately, CPIs have shown globally unsatisfactory results. The aim of this manuscript is to critically review the results from early-phase trials with CPIs in terms of safety and activity, discuss the possible reasons for disappointing results and the new therapeutic approaches to improve patient outcomes.

## 1. Background

Epithelial ovarian cancer (EOC) is the second most common gynecological malignancy with more than 21,750 estimated new cases in US and over 13.900 deaths in 2020 [1].

Most patients (over 70%) are diagnosed with advanced-stage disease and have a five-year survival rate of 29% [2,3] despite multimodal treatment including optimal debulking surgery (no residual tumor) followed by platinum-based chemotherapy (with or without bevacizumab) being the mainstay of initial treatment, although most patients relapse with chemoresistant disease [3,4,5,6,7,8,9,10]. Recently, several inhibitors of the enzyme poly ADP ribose polymerase (PARPi) have been approved in EOC: Olaparib for BRCA-mutated patients as maintenance after first-line chemotherapy and at platinum-sensitive relapse; Niraparib and Rucaparib at platinum-sensitive relapse regardless BRCA status. A progression-free survival (PFS) improvement was observed for all drugs but, to date, with no impact on overall survival (OS) [11,12,13,14,15,16,17] 

Starting from this background, there is clearly a need to develop new therapies. Substantial evidence indicates that EOCs express a multitude of known tumor-associated and mutational antigens (TAAs or neo-antigens, respectively), and a proportion of tumors are infiltrated by intraepithelial tumor-infiltrating lymphocytes (TILs), which correlate with improved survival [18,19,20]. Furthermore, the expression of genes and molecular patterns associated with the immune response identified by The Cancer Genome Atlas Network are associated with longer OS [21,22,23,24].

However, despite the success of immunotherapy in other malignancies such as in melanoma, non-small cell lung cancer (NSCLC) and urothelial cancers [25,26], the use of antibodies inhibiting the immune checkpoint programmed cell death (PD-1) or its ligand (PD-L1) obtained modest results in EOC so far, with median response rates of 10% up to 15% [18,19,20,27]. 

Interestingly, the combination of the anti-PD1 nivolumab and the anti-lymphocyte-associated protein 4 (anti-CTLA4) ipilimumab showed promising results in platinum-resistant EOC at six-month interim analyses with an overall response rate (ORR) of 34% (doubling the results of nivolumab monotherapy). However, final results are still awaited [28].

As a consequence, no immunotherapeutic agent has obtained regulatory approval for EOC thus far.

## 2. PD1/PD-L1

Immune checkpoint regulators are important modulators of the immune system.

These molecules play a central role to control self-tolerance, autoimmunity and regulate tissue damage induced by immune responses. 

The most studied immune checkpoints inhibitors (CPIs) are those involving PD1/PD-L1 and CTLA-4/CD80/CD86 pathways. 

PD-1 is a cell surface co-inhibitory receptor member of the CD28/CTLA-4 family. Physiologically, it is mainly expressed on lymphocytes but also in monocytes and natural killer T cells, following their activation. After binding to its ligands, PD-1 receptor inhibits CD8+ T-cell proliferation and activation and modulates interferon-γ (IFN-γ), tumor necrosis factor-α (TNF- α) and IL-2 production. PD-1 is also highly expressed in regulatory T cells (Tregs), and the binding between PD-1 receptor and its ligand increases Tregs suppressive activity [29,30].

The expression of PD-1 ligands in both tumor cells and tumor-associated macrophages is one of the main mechanisms leading to immune evasion [31]. In this context, the inhibition of the binding between PD-1 and CTLA-4 with its receptors may improve the cytotoxic CD8+ T-cell effectiveness resulting in a higher antitumor activity.

## 3. Early-Phase Clinical Trials in EOC

A better understanding of the immune checkpoint pathways paved the way for the use of CPis in several malignancies. Early-phase trials recruiting EOC patients have been published in the last few years. We will discuss the main features and results below.

As for PD-L1 inhibitors, results from two phase I trials have been reported. 

In the first one, atezolizumab activity and safety were assessed in patients with recurrent uterine (15 cases) and ovarian (12 cases) cancer. In the EOC cohort, 11 patients (91.7%) experienced any grade of treatment-related AEs; of these, two patients had grade 3 events. Nine patients were evaluable for response with an ORR of 22.2% and a DCR of 22.2%. A PD-L1 ≥5% expression on tumor-infiltrating immune cells expression was observed in eight patients, and two of them experienced an objective response (OR) [32].

In the phase Ib study JAVELIN solid tumor, avelumab showed a safe profile in 125 patients with recurrent or refractory EOC. Nine patients (7.2%) had grade 3–4 treatment-related AEs, and 21 patients (16.8%) experienced immune related events of any grade. Data on activity showed an overall response rate (ORR) of 9.6% with only 1 complete response (CR) and a disease control rate (DCR) of 52%. In this study, the expression of PD-L1 did not correlate with the clinical response [33].

As for PD-1 inhibitors, pembrolizumab had a manageable safely profile in a phase Ib study (KEYNOTE-028). This study recruited 26 patients with recurrent OC and PD-L1 expression. Nineteen patients (73.1%) experienced any grade treatment-related AEs, above all G1 and 2, and the most frequent were arthralgia (19.2%), nausea (15.4%) and pruritus (15.4%). Only one G3 AE (transaminase elevation) was registered, and no deaths or discontinuations due to AEs were reported. ORR was 11.5% (3 patients) in this cohort [34]. 

On the basis of these results, pembrolizumab activity was further evaluated in KEYNOTE-100, the largest phase II trial in this setting [35]. It recruited 376 patients with recurrent OC, divided in two cohorts: cohort A, including women that received more than one and less than three prior lines with a platinum-free interval (PFI) or treatment-free interval (TFI) between 3 and 12 months; and cohort B, including heavily pretreated patients (four to six prior lines) with a PFI or TFI longer than 3 months.

Overall, an OR was achieved in 30 patients (8%) with a DCR of 37% in the combined cohort. Specifically, ORR was 7.4% in cohort A and 9.9% in cohort B. Median progression-free survival (PFS) was 2.1 months in both cohorts, with a median overall survival (OS) not reached in cohort A and of 17.6 months in cohort B. The expression of PD-L1 was evaluated using a combined positive score (CPS) that is “the fraction of PD-L1 staining cells (both tumor cells, and immune cells) over the total number of viable tumor cells”. In patients with CPS <1, ORR was 5.0%, whereas it was 10.2% for CPS ≥1 and 17.1% for CPS ≥10 patients. 

Toxicity profile was consistent with KEYNOTE-028; any grade AEs were recorded in 73.1% of patients with 19.7% of them with grade 3–5 AEs.

Another phase II study [36] evaluated the activity and safety of nivolumab (intravenous infusion every 2 weeks at a dose of 1 or 3 mg/kg) in 20 patients with platinum-resistant EOC. It showed a best overall response rate (BOR) of 15% and a DCR of 45%. Median PFS was 3.5 months (95% CI, 1.7 to 3.9 months), and the median OS was 20.0 months.

Grade 3–4 treatment-related AEs occurred in 40% of cases, and two patients experienced severe AEs.

Moreover, no relationship was observed between response to nivolumab and the expression of PD-L1, suggesting, once again, a limited role of this biomarker in assessing response to therapy. 

A summary of the already published data from phase 1 and 2 studies exploring CPIs in OCs is shown in Table 1.

## 4. Adverse Events in Ovarian Cancer Trials

Of all the adverse events (AEs) related to immunotherapy, the most common are fatigue, gastrointestinal, endocrine and dermatological events. Furthermore, although less frequently, neurological, cardiological, pulmonary and renal toxicities are being reported. Overall, 75% of patients treated with ipilimumab experience AEs (of any grade), while in the case of PD/PDL-1 inhibitors, AEs are reported in 30% of cases [37,38]. A recent large retrospective analysis showed an incidence of fatal outcomes in 0.36% of patients treated with PD1 inhibitors and in 1.23% of patients treated with combined therapy (PD1 inhibitors plus anti CTLA-4). The adverse effects with the highest mortality rate are colitis, pneumonia and myocarditis [39,40]. 

The toxicity data of immunotherapy in the EOC treatment are preliminary and come only from phase 1 and phase 2 studies with a relatively low number of recruited patients [32,33,34,35,36]. In these studies, the main side effects experienced by patients are fatigue (7%–41.7%), nausea and/or vomiting (11.5%–25%), hypothyroidism (7.710.6%) and arthralgia (16.7%–25%). Cardiotoxicity is a rare AE during therapy with immune checkpoint inhibitors (CPIs), but surprisingly in the clinical trial UMIN000005714 [36], 6 patients out 20 with arrhythmia, without evidence of myocarditis, were reported. 

Moreover, the KEYNOTE-100 study reported a discontinuation rate of 5.1% due to treatment-related AEs [35]. Despite a strong preclinical rationale for the use of CPIs in EOC, the results of these studies show an overall low response rate at the expense of a certain percentage of AEs. Furthermore, there are currently no available data on the quality of life with these treatments in EOC patients.

## 5. What Can We Do to Improve the Outcomes of Immune Checkpoint Inhibitors?

### 5.1. Better Patient Selection 

#### 5.1.1. Predictive Biomarkers

A crucial point in immuno-oncology, not only for EOC, is to find reliable biomarkers in order to identify the patients who will respond to ICIs. 

Several biomarkers have a higher level of validation such as PD-L1 expression on cancer cells and microsatellite instability (MSI); others, such as BRCA mutation status and tumor mutation burden (TMB) showed more often contradictory results [41].

#### 5.1.2. PD-L1 Assessment

A positive correlation between a high expression of PD-L1 on tumor cells and the clinical response to immunotherapy has been observed in several cancers, most notably LC and urothelial cancer [42]. Unfortunately, these results were not confirmed in EOC by the study of Hamanishi et al., reporting a 68% rate of PD-L1 expression in 70 patients. Patients with a higher expression of PD-L1 were found to have a significantly poorer prognosis compared with patients with lower expression. The five-year survival rates for patients with high-expressing versus low-expressing PD-L1 tumors were 52.6% ± 7.7% versus 80.2% ± 8.9% (*p* = 0.016).

Failure to use PD-L1 as a marker of response to immune CPIs may be related to the following reasons: the method for detecting and measuring the expression of PD-L1, the identification of a cut-off level for positivity, the predictive value of PD-L1 based on the type of cell expression (tumor, lymphocyte, dendritic cell, macrophage), the impact of tumor heterogeneity on the predictive value of PD-L1 and the evaluation of the PD-L1 expression on the primary tumor versus the recurrence.

Interestingly, a better correlation was observed when the PD-L1 expression was measured as combined positive score (CPS), defined as the number of PD-L1-positive cells (tumor cells, lymphocytes, and macrophages) divided by the total number of viable tumor cells and multiplied by 100 [43]; however, despite a higher ORR in patients with CPS >10, this result is comparable with single-agent chemotherapy [44].

Moreover, PD-L1 assessment is even more complex if we consider that, currently, different anti-PD-L1 antibody clones with differences in specificity, sensitivity and possible cross-reactivity between are commercially available. BRCA1/2-mutated EOCs show a higher mutational load and a unique mutational signature with a significantly increased number of TILs, as well as elevated PD-1 expression or PD-L1 expression in tumor-associated immune cells compared to homologous recombination (HR)-proficient tumors.

#### 5.1.3. Microsatellite Instability

Genomic instability, a hallmark of cancer, is generally characterized by DNA mismatch repair (MMR) defects, which lead to MSI. Maintenance of genomic stability ensures the inheritance of a complete copy of genetic material in the daughter cells. Moreover, during replication, cells may develop multiple forms of mutations in several genes, such as chromosomal rearrangements, as well as a gain or a loss of part(s) of or the entire chromosome. Repetitive sequences of 1–6 nucleotide base pairs in DNA are known as microsatellites. In addition, alterations in microsatellites are an important form of genomic instability, referred to as MSI. These tandem repeat sequences are dispersed across the genomes of eukaryotes, usually in noncoding regions. Inactivation of the MMR system results in mutations, particularly highly repetitive sequences. Additionally, the distribution of microsatellites throughout the genome leads to MSI [45]. The reported prevalence of MSI-H status (defined by instability in two or more markers studied) in unselected OC has ranged from 13% to 37% [46].

MMR-deficient tumors exhibit a high expression of pro-inflammatory genes, favoring the migration and activity of CD8 + T cells [47]. Several studies suggest a better prognosis in patients with MMR deficiency treated with immune CPIs than in MMR proficiency patients.

Even if only in a minority of EOC cases are deficient in MMR [48,49], it is worth it to note that the Food and Drug Agency (FDA) granted anti-PD1 approval (Pembrolizumab) for MMR-deficient cancers regardless of histology.

#### 5.1.4. BRCA Status

BRCA status is a well-established predictor of response to PARPs, and, as already mentioned, tumors harboring HRD have a higher predicted neo-antigen load and higher TIL infiltration [50].

However, clinical data show that response to ICI monotherapies is rare in BRCA-mutated patients as well.

BRCA and homologous recombination deficiency (HRD) status were also evaluated in the KEYNOTE-100 study, but no differences were observed between responders and non-responders [35]. However, in a small series of mutated BRCA patients with recurrent EOC, the use of salvage therapy with nivolumab resulted in an ORR of 67% (4 of 6 patients) [51]. 

BRCA status as a response marker has also been evaluated in the TOPACIO/KEYNOTE-162 (Niraparib in combination with Pembrolizumab) and in MEDIOLA (Durvalumab in combination with Olaparib) studies, but preliminary data did not show a correlation between the BRCA mutation and the response to therapy [52,53].

Further data are needed to better understand the role of BRCA as a marker of response to immunotherapy in the EOC. Moreover, consistency in terminology and thresholding is required (when referring to HRD status) in order to optimally use such biomarkers in clinical practice, as well as improved assays that optimize their predictive value [54].

#### 5.1.5. Histology

Data presented from the above clinical studies were obtained from relatively small and heterogeneous cohorts of patients. Most of them were heavily pre-treated, and different immune CPIs at different dosages were used, so we are still far from understanding which is the best drug and the best therapeutic regimen.

An interesting observation derives from the study by Hamanishi et al., which reports a complete response in a 60 year old patient with recurrent clear-cell OC treated with nivolumab [36]. Furthermore, Matulonis et al. [35] also reported a positive trend in the response rate of patients with clear-cell OC treated with pembrolizumab.

These cases remain anecdotal observations; however, in clear-cell renal tumors immunotherapy has shown a significant improvement in OS [55]. There is also evidence showing that clear-cell tumors showed similar genetic profiles [56]; therefore, it is possible to hypothesize that this subset of patients could benefit from immunotherapy.

More intriguing, according to the status of TIL infiltration, tumors could be histologically categorized as “inflamed/hot tumors” or “non-inflamed/cold”. The first ones are characterized by the presence in the tumor bed of a high density of CD8+ T cells [57,58], and such patients could benefit from therapies acting on T cell checkpoints involved in immune tolerance. On the contrary, cold tumors are characterized by the absence of T cells in tumor beds and are generally affected by a failure in T cell priming, reflecting the need of strategies that could deliver autologous/allogenic effector cells into the cancer. A third phenotype, defined as “immune-excluded”, is characterized by modification of the tumor microenvironment (TME) and the presence of inhibitory cells that retain CD8 T cells from entering the tumor islets, even if they are present in the stroma. Such patients could benefit from strategies whose aim is to increase infiltrations of tumors by immune effector cells such as T cell trafficking modulators, epigenetic modulators, TME remodeling molecules and radiation therapy [59].

#### 5.1.6. Tumor Mutation Burden

A recent analysis in the KEYNOTE-100 showed no statistically significant differences in HRD status among responders and non-responders and the absence of association between BRCA status and responses. Interestingly, tumor mutation burden (TMB) and T cell-inflamed gene expression profile (GEP) were independently predictive of response and demonstrated low correlation, suggesting that they capture distinct features of neo-antigenicity and T cell activation.

In the recent review by Lu et al. [41], multiplex immunohistochemistry/IF and multimodality biomarker strategies appear to be associated with improved performance over PD-L1 IHC, TMB, or GEP alone. Further studies with composite approaches and a larger number of patients will be required to confirm these findings to determine the most predictive combinations according to tumor type.

## 6. Novel Combinations

### 6.1. PARP Inhibitor and Immune Checkpoint Inhibitor

PARP are a family of enzymes that participate in various cellular processes adding poly (ADP-ribose) chains onto target molecules (a process known as PARylation).

Particularly, PARP1 is mostly associated with DNA damage repair, which generates nearly 90% of poly (ADP-ribose) chains after the induction of DNA damage. This DNA repair process is fundamental in cells that have lost homologous repair mechanisms. Indeed, PARPis are particularly effective in cancer cells that have mutations of the genes BRCA1 or BRCA2 [60]. In fact, PARPi interferes with homologous DNA damage repair, increases the mutational load in tumor cells and, in mouse models with mutations in BRCA genes, activates interferon-mediated pathways by acting in synergy with the inhibitors of immune checkpoint inhibitors [61]. Moreover, in the EOC with BRCA1/2 mutation, higher PD-1 expression in TILs has been reported compared with homologous recombination-proficient tumors. 

Based on this rationale, the association between PARPis and immune CPIs is a potentially successful approach for EOC therapy. Several clinical trials testing this association are currently ongoing (Table 2).

As for the first-line setting, three ongoing studies are evaluating a combination of PARPi and CPis in front-line and as maintenance therapy after platinum-based first-line therapy (KEYLYNK-001, FIRST, ATHENA), but no preliminary data are yet available.

In the recurrent, platinum-sensitive setting, an open-label, phase II basket study (MEDIOLA, NCT02734004) evaluated safety and activity of Olaparib and Durvalumab association in germline BRCA-mutated EOC patients. Preliminary results showed an 81% DCR at 12 weeks and a 63% ORR, with good tolerability profile [62]. 

An ongoing phase III trial is evaluating a combination of niraparib and pembrolizumab in heavily pretreated platinum-sensitive recurrent OC as maintenance after platinum-based chemotherapy.

In patients with platinum-resistant EOC, a phase I-II study (TOPACIO/Keynote-162, NCT02657889) demonstrates that Niraparib combined with Pembrolizumab achieves an ORR of 25% in the overall population with 45% in the subgroup with BRCA 1/2 mutations [63]. 

Moreover, an interesting phase I/II study is ongoing evaluating the combination of the anti-CTLA-4 (Tremelimumab) and Olaparib in BRCA mutated recurrent OCs (NCT0251725).

### 6.2. Anti-Angiogenic Drugs and Immune Checkpoint Inhibitor

Another possibility to increase the effectiveness of immune CPIs is to combine them with angiogenesis inhibitors.

In preclinical models, a synergistic effect was observed between the alteration of tumor angiogenesis and the increase in the immune response.

Shrimali et al. have shown that the "normalization" of the tumor vascular architecture by inhibiting VEGF as well as improving the effectiveness of chemotherapy improves tumor infiltration by the adoptively transferred T cells [64]. Similar results were reported by Dings et al., which showed significant inhibition of tumor growth using adjuvant anti-angiogenic therapy in combination with T-cell transfer [65]. 

Studies are currently underway to evaluate the efficacy and the safety of anti-angiogenic drugs in combination with immune CPIs.

In particular, we have no clinical data on first-line setting, but an ongoing phase III study is recruiting patients with newly diagnosed high-risk OC, evaluating pembrolizumab and bevacizumab combination (GOG3015). 

In patients with recurrent disease, the results of a single-arm phase 2 study on 38 patients (18 with platinum-resistant disease and 20 with platinum-sensitive) with recurrent OC that evaluated a combination of nivolumab and bevacizumab have been recently reported. The ORR was 40.0% in platinum-sensitive cohort and 16.7% in platinum-resistant patients.

About 89% of patients experienced at least one treatment AEs of any grade, while 23.7% experienced ≥3 AEs. The results of this study suggest an activity of this combination in platinum-sensitive EOC [66]. 

As for platinum-resistant patients, a phase 1 study tested durvalumab plus cediranib in 14 patients with a 55% ORR; however, frequent severe AEs were reported for this association (hypertension and diarrhea) [67]. 

In this setting an interesting three-arm phase III study recruited platinum-resistant ROC randomized patients to bevacizumab alone or in combination with atezolizumab or in triplet with atezolizumab and aspirin. The primary endpoint is 6 months PFS. Recruitment is closed and results are awaited.

### 6.3. Triplets Including PARPi, Anti-Angiogenic Drugs and an Immune Checkpoint Inhibitor

A powerful strategy may combine CPIs with both PARPis and immune-checkpoint inhibitors. Clinical data will be provided by DUO Phase III study that recruits Stage III-IV OC. Patients will receive platinum-based therapy with bevacizumab and durvalumab followed by maintenance bevacizumab, durvalumab plus olaparib or placebo in arms A and B respectively. 

A complete list of ongoing phase III trials exploring ICIs and PARPIs and/or anti-VEGF combinations are listed in Table 2, while the preliminary results of drug combinations are reported in Table 3.

### 6.4. Radiotherapy and Immune Checkpoint Inhibitor

There is a potential rationale for the use of radiotherapy plus immune CPIs in patients with advanced EOC. Low doses of radiation therapy can induce DNA damage and trigger an “in situ vaccination” process by activating antigen-presenting cells and enhancing the cell-mediated immune response. Moreover, radiotherapy can trigger the apoptosis of Treg lymphocytes, further promoting the activity of cytotoxic T-cells [68].

In this context, radiotherapy can enhance the immune response against EOC and the effects of immunotherapy. Recently, some authors have demonstrated a role of radiotherapy alone in oligometastatic EOC [68,69,70], and some clinical studies are evaluating the efficacy and safety of checkpoints combined with radiotherapy. The most effective radiotherapy dosage and fractionation to obtain the activation of the immune response are still to be defined. In addition, pelvic and abdominal radiotherapy may have important gastrointestinal and urinary toxicities [68,71,72].

### 6.5. Chemotherapy and Immune Checkpoint Inhibitor

Strong preclinical data suggest that chemotherapy could improve the activity of CPIs. Indeed, adding carboplatin to PD-L1 antibodies increases CD4 and CD8 T lymphocytes reducing T reg cells, while the addition of paclitaxel to a PD-L1/PD-1 inhibitor increases PD-L1 expression and survival of mice [73].

Based on these premises, two phase III trials evaluated the combination of avelumab to standard chemotherapy in frontline treatment (JAVELIN 100) and in platinum-resistant disease (JAVELIN 200). 

JAVELIN 100 is a phase III randomized trial that evaluated avelumab both in combination or only as maintenance therapy after carboplatin and paclitaxel in first line. The study was prematurely terminated on the basis of the results of a planned interim analysis that showed futility of efficacy in Dec 2019 (NCT02718417).

JAVELIN 200 recruited 566 patients with platinum-resistant disease and no more than 3 prior therapies, randomizing them to pegylated liposomal doxorubicin (PLD), avelumab or both of them. Primary end points were PFS and OS. Combination of PLD and avelumab did not prolong the median PFS (3.5 months *vs* 3.7 months) or the median OS (13.1 months *vs* 15.7 months) (hazard ratio, 0.89; 95% CI, 0.74–1.24) [74].

These results suggest that a combination of chemotherapy and CPIs might not be the most successful strategy for OC patients. The role of chemotherapy with immunotherapy needs further investigation, but it could play a role in combination with adoptive therapies (that are based on the infusion of autologous or allogenic immune cells that actively kill cancer cells) [75].

## 7. Conclusions

Immunotherapy has dramatically changed the prognosis of several cancers such as NSCLC melanoma, bladder and kidney cancer. Although CPIs use in EOC is strongly sustained by preclinical rationale, clinical results from early development trials are largely disappointing so far. 

A better selection of patients based on tumor histology, BRCA/HRD status, and previous treatments may improve these results.

The main objective is to personalize immunotherapy in order to efficiently cure EOC patients, avoid unnecessary toxicities and reduce the costs for the healthcare systems [76]. Improvement of academic studies and translational research is crucial to improve survival of EOC patients.

## Figures and Tables

**Table 1:** Results from phase 1 and 2 studies exploring efficacy and safety of single-agent CPIs in recurrent OC.

Study	Drug	Phase	Clinical Setting	Primary End Point	Number of Included Patients	Most Common Adverse Events Reported (Any Grade)	ORR	PFS (Months)	OS (Months)	Ref
JAVELIN solid tumors, NCT01772004	Avelumab (Anti PD-L1)	1	Recurrent or refractory disease OC, FTC or PPC	DLTs, BOR	125	Fatigue (14%), diarrhea (12%), nausea (11%)	9.6%	10.2	11.2	Disis et al [33]
NCT01375842	Atezolizumab (Anti PD-L1)	1	Recurrent or metastatic EOC or advanced/recurrent uterine cancer	DLTs, MTD, RP2D, %AEs	12	Fatigue (33%), chills (33%), pain (33%), pyrexia (33%)	22.2%	2.9	11.3	Liu et al [32]
KEYNOTE-028, NTC02054806	Pembrolizumab (Anti PD-1)	1b	Recurrent advanced OC, FTC or PPC	BOR	26	Arthralgia (19%), nausea (15%), pruritus (15%)	11.5%	1.9	13.8	Varga et al [34]
KEYNOTE-100, NCT02674061	Pembrolizumab (Anti PD-1)	2	Recurrent OC, FTC or PPC	ORR	376	Fatigue (34%), nausea (15%), decreased appetite (11%), hypothyroidism (11%)	8.0%	1.9	13.8	Matulonis et al [35]
UMIN00005714	Nivolumab (Anti PD-1)	2	Advanced or relapsed, platinum-resistant OC	ORR, PFS, OS	20	AST increased (40%), hypothyroidism (40%), lymphocytopenia (35%)	15%	3.5	20.0	Hamanishi et al [36]

BOR: best overall response, CPIs: checkpoint inhibitors, DLTs: dose-limiting toxicities, FTC: fallopian tube cancer, OC: ovarian cancer, ORR: objective response rate, OS: overall survival, MTD: maximum tolerated dose, PPC: primary peritoneal cancer, PFS: progression-free survival, RP2D: recommended phase II dose.

**Table 2:** Ongoing phase III trials exploring combination of CPIs and PARPIs and/or anti-VEGF drugs.

Study	Setting		Enrollment	Arms	Primary Endpoints	Current Status
AGO/DUO-ENGOT Ov46; NCT03737643	Front line and maintenance	Stage III-IV OC, FTC, or PPC. UPS of IDS.	1056 patients	Carboplatin-taxol + bevacizumab + placebo followed by +bevacizumab + placebo + placebo Carboplatin-taxol + bevacizumab + durvalumab (anti PD-L1) followed by bevacizumab + durvalumab + placebo Carboplatin-taxol + bevacizumab + durvalumab followed by bevacizumab + durvalumab + olaparib	PFS in non-tBRCA mutated	Recruiting
KEYLYNK-001/ENGOT-ov43; NCT03740165	Front line and maintenance	Stage III-IV OC, FTC, or PPC, all hystotypes excluding mucinous, germ cell, or borderline tumors. UPS of IDS.	1086 patients	Carboplatin-taxol + placebo followed by + placebo Carboplatin-taxol + pembrolizumab (anti PD-1) followed by pembrolizumab+ placebo Carboplatin-taxol + pembrolizumab followed by pembrolizumab+ olaparib	PFS and OS	Recruiting
GINECO/FIRST ENGOT Ov44; NCT03602859	Front line and maintenance	Stage III-IV OC, FTC, or PPC, all hystotypes excluding mucinous, germ cell, or borderline tumors. UPS of IDS.	912 patients	Carboplatin taxol + placebo followed by placebo Carboplatin taxol + placebo + followed by placebo+ niraparib Carbo-tax + dostarlimab (anti TSR042) followed by+ dostarlimab (anti PD1) +niraparib	PFS	Recruiting
ATHENA GOG3020/ENGOT Ov45; NCT03522246	Maintenance after front line	Stage III-IV OC, FTC, or PPC. Completed first-line platinum-based chemotherapy and surgery with a response. UPS or IDS.	1012 patients	Rucaparib + nivolumab (anti PD1) Rucaparib + placebo Nivolumab-placebo Placebo + placebo	PFS	Recruiting
GOG3015/ENGOT OV39; NCT03038100	Front line	Stage III-IV OC, FTC, or PPC with macroscopic residual disease postoperatively or neoadjuvant therapy followed by IDS.	Estimated 1300 patients	Carboplatin-taxol + bevacizumab Carboplatin-taxol + bevacizumab + atezolizumab (anti PD-L1)	PFS and PFS in PD-L1 + subpopulation; OS and OS in PD-L1 + subpopulation	Active
ENGOT-Ov41/GEICO 69-O/ANITA; NCT03598270	Recurrence Platinum sensitive	PFI > 6 months and 2 prior lines of chemotherapy. The last line of chemotherapy should have included platinum. BRCA status known	414 patients	Carboplatin combo + niraparib Carboplatin combo + niraparib + atezolizumab	PFS	Recruiting
ATALANTE/ENGOT OV29; NCT02891824	Recurrence Platinum sensitive	PFI > 6 months and 2 prior lines of chemotherapy. The last line of chemotherapy should have included platinum.	600 patients	Carboplatin combo + bevacizumab Carboplatin combo + bevacizumab + atezolizumab	PFS	Active
EORTC-1508, NCT02659384	Recurrence Platinum resistant	Platinum resistant EOC, FTC or PPC. Any number of platinum-based chemotherapy lines, but a maximum of 2 previous non-platinum containing lines. Prior treatment with bevacizumab or other targeted agents	Estimated 160 patients	Bevacizumab Bevacizumab + atezolizumab Bevacizumab + atezolizumab + aspirin	PFS at 6 months	Closed to recruitment

OC: ovarian cancer, FTC: fallopian tube cancer, PPC: primary peritoneal cancer, UPS: upfront primary surgery, IDS: interval debulking surgery, PFS: progression-free survival, OS: overall survival.

**Table 3:** Results in trials exploring combination therapy (ICIs + PARPi or anti-VEGF).

Study	Patient Selection	Number of Patients	Treatment	Most Common Adverse Events Reported any Grade	DCR	ORR
MEDIOLA, NCT02734004	Recurrent platinum-sensitive OC, FTC, PPC with germline BRCA mutations in second-line or later therapy	32	Durvalumab (anti PD-1) + olaparib Durvalumab + olaparib + bevacizumab	Hypothyroidism (15%); cutaneous rash (12%)	81% at 12 weeks	63% at 12 weeks
TOPACIO/Keynote-162, NCT02657889	Recurrent OC, FTC, PPC with germline BRCA mutations	60	Pembrolizumb (anti PD-1) + niraparib	Fatigue (53%), nausea (42%), anemia (36%), constipation (36%)	45%	25%
NCT02484404	Recurrent OC, FTC, PPC received least two prior platinum-containing regimens, platinum resistant or refractory	26	Durvalumab + olaparib (N = 12) or cediranib (n = 14)	Olaparib arm: fatigue (75%), nausea (58%), Abdominal pain (42%) (any grade); Cediranib arm: hypertension (86%), diarrhea (72%) (any grade)	Not reported	17% (Olaparib arm); 55% (Cediranib arm)
NCT02873962	Recurrent OC, FTC, PPC. All histotypes. Platinum-resistant or platinum-sensitive disease.	38	Nivolumab (anti PD-1) + bevacizumab	Fatigue (47%), headache (29 %), myalgia (29%), serum amylase level increase (29%) (any grade).	Not reported	40.0% in platinum-sensitive (patients) 16.7% in platinum-resistant (patients)

ICIs: immune checkpoint inhibitors, PARPi: inhibitors of poly (ADP-ribose) polymerase, VEGF: vascular endothelial growth factor, OC: ovarian cancer, FTC: fallopian tube cancer, PPC: primary peritoneal cancer, DCR: disease control rate, ORR: objective response rate.
